# Direct nn-Scattering Measurement With the Pulsed Reactor YAGUAR

**DOI:** 10.6028/jres.110.029

**Published:** 2005-06-01

**Authors:** G. E. Mitchell, W. I. Furman, E. V. Lychagin, A. Yu. Muzichka, G. V. Nekhaev, A. V. Strelkov, E. I. Sharapov, V. N. Shvetsov, Yu. I. Chernuhin, B. G. Levakov, V. I. Litvin, A. E. Lyzhin, E. P. Magda, B. E. Crawford, S. L. Stephenson, C. R. Howell, W Tornow

**Affiliations:** North Carolina State University, Raleigh NC, USA 27695-8202; Triangle Universities Nuclear Laboratory, Durham NC, USA 27708-0308; Joint Institute for Nuclear Research, 141980 Dubna, Russia; Russian Federal Nuclear Center–All-Russian Research Institute of Technical Physics, P.O. Box 245, 456770 Snezhinsk, Russia; Gettysburg College, Box 405, Gettysburg PA, USA 17325; Duke University and Triangle Universities Nuclear Laboratory, Durham NC, USA 27708-0308

**Keywords:** charge-symmetry breaking, nn-scattering, pulsed reactor

## Abstract

Although crucial for resolving the issue of charge symmetry in the nuclear force, direct measurement of nn-scattering by colliding free neutrons has never been performed. At present the Russian pulsed reactor YAGUAR is the best neutron source for performing such a measurement. It has a through channel where the neutron moderator is installed. The neutrons are counted by a neutron detector located 12 m from the reactor. In preliminary experiments an instantaneous value of 1.1 × 10^18^/cm^2^s was obtained for the thermal neutron flux density. The experiment will be performed by the DIANNA Collaboration as International Science & Technology Center (ISTC) project No. 2286.

## 1. Introduction

After many years of conflicting reports for the neutron-neutron ^1^S_0_ scattering length *a*_nn_, by the late 1990s the results appeared to have converged to consistent numbers: *a*_nn_ = −18.55 ± 0.05 (stat) ± 0.3 (syst) fm [1] from the ^2^H(π^−^, γn)n reaction, and *a*_nn_ = −18.7 ± 0.3 (stat) ± 0.6 (syst) fm [2] from the ^2^H(n, nn)p reaction. Comparison with the well established value of the proton-proton scattering length *a*_pp_ = −17.3 ± 0.005 (stat) ±0.4 (syst) fm indicates charge-symmetry breaking (CSB).

However, a recent investigation of the neutron-neutron and neutron-proton final-state interaction in the n-d breakup reaction revived this issue. The *a*_nn_-value of −16.27 ± 0.40 fm was obtained in Ref. [3] for the ^2^H(n, np)n reaction, where the total uncertainty consists of about equal parts of statistical and systematic errors. This latest result differs from that of Ref. [2] by almost 4 *σ*, and renews the long-standing controversy regarding the conclusion on charge-symmetry breaking. The possible solution is a direct measurement of the nn-scattering length. Such a measurement has been never performed. Here we describe specifics of the direct nn-scattering measurement which is under preparation at the unique aperiodic pulsed reactor YAGUAR.

## 2. General Scheme of the Experiment

In effective-range theory, the singlet scattering cross section *σ*_s_ is defined by the scattering length *a*_nn_ as

$$\sigma_{s}=4\pi a_{\mathrm{nn}}^{2}\;\text{at}\;k\to 0,$$ (1)

where *k* is the neutron wave number. For thermal neutrons the zero effective-range approximation works well. The effective thermal neutron cross section *σ* measured with unpolarized neutrons is a statistical sum of the singlet *σ*_s_ and triplet *σ*_t_ cross sections

$$\sigma =\frac{1}{4}\sigma_{s}+\frac{3}{4}\sigma_{t}=\frac{1}{4}\sigma_{s}=\pi a_{\mathrm{nn}}^{2}.$$ (2)

Since the Pauli exclusion principle for identical particles forbids the interaction of two neutrons in the triplet state, the cross section *σ*_t_ is expected to be zero, and the measured cross section *σ* is equal to $\pi a_{\mathrm{nn}}^{2}$.

Proposals for a direct measurement of the nn-scattering length have a long history; detailed references are provided in Ref. [4]. None of these proposals has been implemented. Except for the proposed use of underground nuclear explosions, most of the proposals are similar. The neutrons collide in a neutron interaction chamber, which we denote as the nn-cavity, and the scattered neutrons are detected externally. In such arrangements the “target” and the “beam” are neutrons produced by the same neutron source. Therefore, the nn scattering intensity is proportional to the square of the neutron flux density, while the background intensity depends linearly on the flux density.

The situation is most favorable at pulsed neutron sources, where the thermal neutron flux density can be very high. It appears that the pulsed aperiodic reactor YAGUAR [5] at Snezhinsk, Russia, fulfills requirements for the nn cross section measurement. This nn project was initially proposed at the Dubna ISINN-VIII meeting [6]. During the fast neutron pulse of 0.68 ms (FWHM) duration, about 10^18^ fast neutrons are generated in the active core. The 40 L volume of the YAGUAR active core contains the water soluted salt UO_2_SO_4_. The solution contains 465 g/L of uranium which is enriched to 90% ^235^U. The effective diameter of the cylindrical active core is 40 cm, with an effective inside diameter of 17 cm. The critical height is about 39 cm, depending on the position of the startup rods. The body of the reactor has a central channel of 15 cm diameter, which in the standard operating mode has startup rods that leave a cylindrical space of 12 cm diameter.

**Fig. 1:**
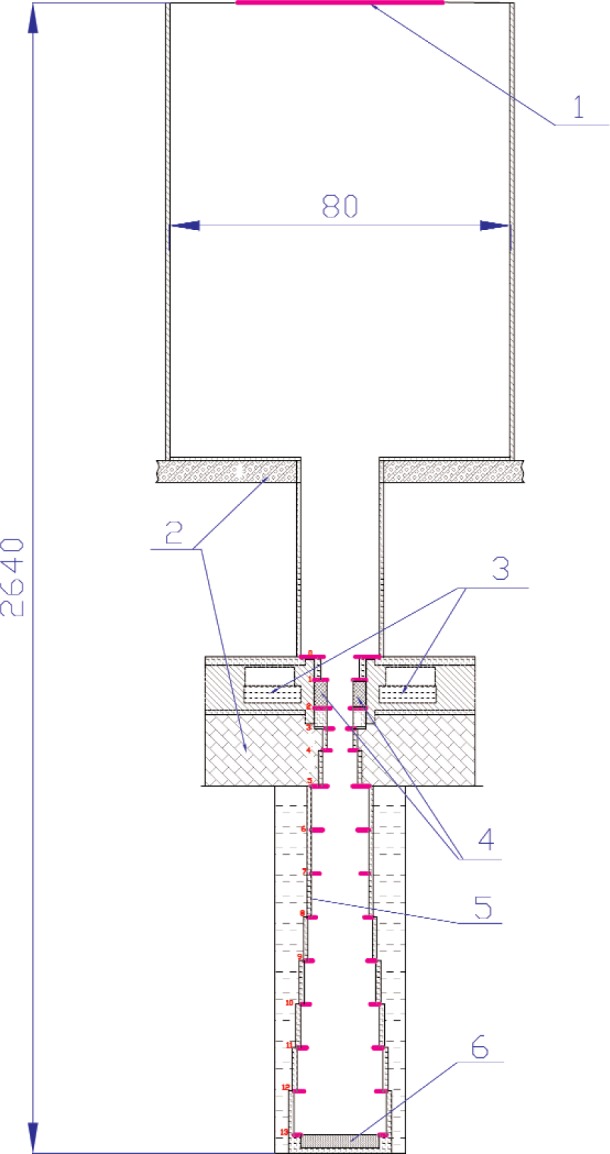
Setup for the nn-scattering experiment. The horizontal scale is ten times greater than the vertical scale. See text for explanation.

A direct measurement of the nn-scattering length is to be performed by the time-of-flight (TOF) method by counting the scattered neutrons arriving after the reactor burst at a detector placed behind special collimators far away from the reactor. If only the colliding neutrons contribute to the detector counting rate, and if the parameters of the neutron field are determined and the geometry is known, then the detector counts (integrated over the thermal neutron part of the TOF spectrum), measure the nn-scattering cross section. The proposed arrangement for the nn-scattering experiment at YAGUAR is shown schematically in Fig. 1. The active core 3 is placed on three supports at 2 m above the floor level. The neutron CH_2_ moderator 4 is inserted inside the channel. An evacuated tube contains a collimation system 5, and the neutron detector 6 is placed at a flight path of about 12 m. The collimation system is designed to screen the source and to minimize background due to neutrons scattered from the walls. The ^3^He absorber 1 reduces neutron scattering from the back wall.

## 3. Neutron Flux

The reactor produces a hard neutron spectrum in the through-channel (average neutron energy 0.91 MeV) that is not suitable for the nn-experiment. We performed calculations for various moderators in the YAGUAR geometry and also performed measurements with various moderators at the reactor YAGUAR. The Monte Carlo modeling [7] was performed using the code MCNP-4 [8]. The cylinder height of 40 cm was fixed and the thickness of the polyethylene cylindrical shell varied.

Neutron activation studies were performed to measure the thermal neutron densities inside cylindrical converters inserted into the YAGUAR reactor channel. The polyethylene converters were fabricated as hollow cylinders of 39.7 cm length and 12.0 cm outer diameter, with different inner diameters. The activation detectors for the absolute flux measurements were gold and copper foils placed in the central plane. One detector set was covered with a 1 mm thick Cd screen. Details of the measurements are given in [4]. The relative distribution of the neutron flux along the axis of the through-channel was also obtained. These measurements, combined with known neutron activation cross sections, determined the fluency, defined as the number of neutrons per cm^2^ per YAGUAR pulse.

The measured neutron fluencies for the four polyethylene converters vary linearly with thickness. This dependence agrees very well with Monte Carlo simulations [7]. With the experimental results for the fluence and using the value Δ*t* = 0.7 ms for the thermal neutron pulse, we conclude that the 3 cm thick cylindrical moderator provides, for the pulse power of 30 MJ, an instantaneous value of 1.1 × 10^18^/cm^2^s for the thermal neutron flux *Φ*_central_ in the central region of the channel.

**Fig. 2:**
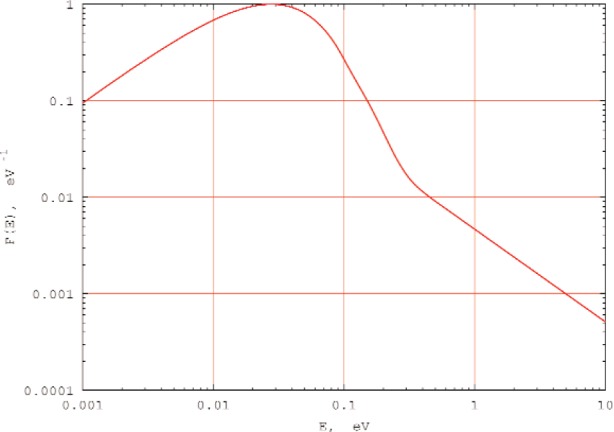
Neutron flux *F*(*E*) versus neutron energy *E* inside the cylindrical cavity with the 2.2 cm thick moderator. The spectrum is normalized to unity at the maximum.

The moderated neutron flux spectrum is expected to be predominantly Maxwellian and to have a 1/*E^n^* epithermal tail with the value of n depending on the moderator thickness. The gold and copper activation data were analyzed to obtain experimental estimates of such parameters as the effective Maxwellian temperature, the relative size of the epithermal tail, and the slope parameter n. The neutron flux spectrum generated with these parameters is shown in Fig. 2 for the 2.2 cm thick moderator. The MCNP modeling of the neutron fluxes for different moderator thicknesses agrees with the experimental activation results. Based on these results we chose the moderator thickness value of 2.5 cm for future nn measurements.

## 4. Simulations

### 4.1 Counting Rates

After nn-scattering in the evacuated moderator channel, some of the scattered neutrons will reach a detector placed at about 12 m from the channel central plane, and will be measured by the TOF method with an effective solid angle *Ω*_eff_. For ideal collimation, direct neutron paths from the moderator and the walls (besides the back wall of the nn cavity) to the detector are excluded. Then the detector counts *N*_nn_ per pulse (integrated over the thermal part of the TOF spectrum) are related to the nn-scattering cross section *σ*_nn_, the average neutron flux density *Φ*_av_, the effective pulse duration Δ*t*, the effective nn-cavity volume *V*, and the most probable velocity *v*_0_ by

$$N_{\mathrm{nn}}=2c_{\mathrm{av}}\frac{\Phi_{\mathrm{av}}^{2}}{v_{0}}\sigma_{\mathrm{nn}}\Delta tV\;\Omega_{\mathrm{eff}}\;(\text{counts/pulse}),$$ (3)

where the constant *c*_av_ is determined by Monte Carlo and analytical calculations for a given moderator geometry and neutron flux distribution. For a pure thermal neutron spectrum and including the CM/LAB transformation of the scattered flux in the direction of the detector, the value of *c*_av_ = 0.83 ± 0.01 [9]. Background considerations and the effects of fast-thermal and fast-fast collisions are discussed in subsections that follow. With a realistic value for the solid angle (*Ω*_eff_ ≃ 5 × 10^−6^), then from Eq. (3) we expect *N*_nn_ ≃ 180 nn-counts in the neutron detector per burst.

### 4.2 Backgrounds

Of paramount importance for the direct nn-scattering measurement is the detector background. Ideally with extensive shielding no slow or fast source neutrons can reach the detector directly. However, in practice one can expect several background components.

The thermal neutron background component from the thermal and epithermal neutrons multiply scattered on collimators will depend on the collimation system. Several collimator options, as presented in [4] and in references therein, were considered, and an estimate of this component was found to be less than 20% even for the simplest two-diaphragm collimation. With the use of Boron-10 coated collimators one can reduce this component considerably. The inevitable scattering from the back wall will be reduced by choosing an optimal geometry and the use of the TOF technique.

Fast neutrons that originate during the reactor pulse are also separated by the TOF measurement. The time interval allocated for detecting thermal neutrons is about 5 ms and is delayed by ≈2 ms relative to the maximum of the reactor pulse. At this time the subcritical reactor continues to emit fission neutrons. During this 5 ms interval about *N* ≈ 10^13^ neutrons from the bottom area of the reactor vessel are directed into the lower hemisphere. These neutrons penetrate partially through shields and can present a background problem. This issue was addressed in Ref. [10]. The Monte Carlo code MCNP4-C with the weight window variance reduction technique was chosen to calculate the neutron transport for a 6 m model of the shield consisting of water, concrete, and earth, with a 6 cm diameter channel lined with boron carbide. Scaled to the real geometry the results obtained correspond to a fast neutron background level of ≈5%.

The background-to-nn signal ratio from neutron scattering on the residual gas depends on the gas pressure and does not depend on the collimation system. At a gas pressure of ≤10^−6^ torr, this background component is estimated to be ≤1%.

Therefore, preliminary calculations on the background issues are promising, and more extensive modeling by transport code experts is underway at Snezhinsk. Direct experimental separation of the nn-signal from the background is also possible, because the nn-scattering intensity is proportional to the square of the neutron flux density, while the background intensity depends linearly on the flux density.

### 4.3 Time-of-Flight Spectra

**Fig. 3:**
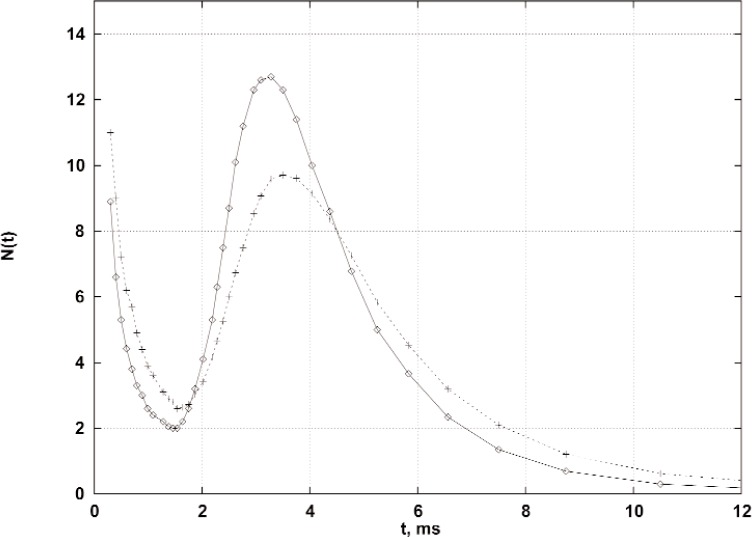
Calculated time-of-flight spectra *N*(*t*) (in relative units) for the 12 m flight path. Full line: the “incident” (before scattering) neutron flux from the 2.5 cm thick cylindrical moderator; broken line: the nn-scattered flux. The spectra are normalized to equal areas.

The nn scattering measurements at the pulsed reactor YAGUAR will be performed by the TOF method. The transformation from the energy spectrum of the incident flux *F*(*E*)d*E* (as shown in Fig. 2) to the TOF spectrum *N(t*) is accomplished by the use of equations *F*(*E*)d*E = N*(*t*)d*t* and d*E* = −2(d*t/t)E.* For a 12 m flight path and 2.5 cm thick cylindrical moderator the simulated “incident” TOF spectrum is shown in Fig. 3 by the full curve. Such a spectrum would be measured by the neutron detector if it were placed as shown in Fig. 1 and the nn cavity filled with a heavy gas such as argon, for which the transformation of the neutron spectrum after scattering can be neglected. The spectrum essentially consists of a Maxwellian part above *t* = 2 ms and a 1/*t*^2^ epithermal part. There is also an intermediate distribution for *t* between 1.6 ms and 2.0 ms. For convenience we refer to the distributions above and below t* = 2 ms as the “slow” and “fast” components. This value of t* is expected to be a cut off value for the experimental TOF spectrum below which the background (not shown in Fig. 3) will increase sharply.

However, for the collision of light particles with equal masses, energy and momentum conservation leads to the well known “step” distribution of final energies extending down to zero and to an anisotropy of scattering in the laboratory (LAB) coordinate system. Questions arise about the shape of the scattered neutron spectrum in the YAGUAR experiment and concerning the size *g* of a possible contribution to the thermal energy region from “fast-slow”, $g_{\mathrm{fs}}^{s}$, and “fast-fast”, $g_{\mathrm{ff}}^{s}$, collisions producing “slow” neutrons. These questions can be answered in two ways: analytically, by invoking a complicated double differential scattering cross section in the LAB system, or numerically, by calculating the collision rates for initial neutron trajectories (for neutrons emitted from the internal cylindrical surface of the moderator), simulating the isotropic angular distribution of scattering in the CM coordinate system, and finally by performing analytical transformation of the scattered velocity vectors to the LAB system in order to follow neutron trajectories to the detector.

For the latter modeling, a new code was written as described in [11]. The TOF spectrum of the scattered neutrons calculated with this code is shown in Fig. 3 by a broken curve while the initial non-scattered spectrum is drawn by the full curve. With these spectra normalized to equal areas the difference in shapes is quite evident. However, the thermal parts of the scattered spectra for cases of the realistic and the Maxwellian inputs remain nearly the same. The code performs a proper analysis of the “fast-fast”, “fast-slow” and “slow-slow” contributions to the thermal part of the realistic spectrum. According to this analysis, the integral of the realistic scattered spectrum above t* changes only slightly as compared to the pure Maxwellian case. The preliminary numbers are: $g_{\mathrm{fs}}^{s}≃6\%,g_{\mathrm{ff}}^{s}\leq 0.1\%$; graphical illustrations are given by Crawford [11].

These issues are relevant to the *c*_av_ value in Eq. (3) which is now expected to change only slightly between the cases of the pure Maxwellian and realistic spectra. Calculations of *c*_av_ for the YAGUAR neutron flux spectrum with an epithermal tail are currently in progress.

## References

[1] Howell CR (1998). Phys Lett B.

[2] González Trotter DE (1999). Phys Rev Lett.

[3] Huhn V, Wätzold L, Weber Ch, Siepe A, von Witsch W, Witala H, Glöckle W (2001). Phys Rev C.

[4] Furman WI (2002). J Phys G: Nucl Part Phys.

[5] Levakov BG, Gorin NV, Kurakov NP, Lukin AV, Lyzhin AE, Nevodnichy NN (1994). Proceedings of the Topical Meeting on Physics.

[6] Bowman CD, Levakov BG, Lyzhin AE, Lychagin EV, Magda EP, Muzichka AYu, Strelkov AV, Sharapov EI, Shvetsov VN (2000). ISINN-VIII, Report E3-2000-192.

[7] Stephenson SL (2003). ISINN-X, Report E3-2003-10.

[8] Briesmeister JF (2000). MCNP™—A General Monte Carlo N-Particle Transport Code, Version 4c, Report LA-13709-M.

[9] Crawford BE (2003). ISINN-X, Report E3-2003-10.

[10] Gueorguiev GP, Howell CR, Mitchell GE, Sharapov EI, Tornow W (2004).

[11] Crawford BE (2004). J Phys G: Nucl Part Phys.

